# Can a Set of Questions after Routine Cataract Surgery Predict Unexpected Findings and Avoid an Unnecessary Follow-Up Visit?

**DOI:** 10.3390/medicina57111144

**Published:** 2021-10-22

**Authors:** Reda Zemaitiene, Ieva Pasiskeviciute, Aiste Varoniukaite, Pijus Pajeda, Andrzej Grzybowski, Dalia Zaliuniene

**Affiliations:** 1 Department of Ophthalmology, Medical Academy, Lithuanian University of Health Sciences, 44037 Kaunas, Lithuania; ieva.pasiskeviciute@gmail.com (I.P.); aiste.varoniukaite@kaunoklinikos.lt (A.V.); pijus.pajeda@kaunoklinikos.lt (P.P.); dalia.zaliuniene@lsmuni.lt (D.Z.); 2 Department of Ophthalmology, University of Warmia and Mazury, 10719 Olsztyn, Poland; ae.grzybowski@gmail.com; 3 Institute for Research in Ophthalmology, Foundation for Ophthalmology Development, 61553 Poznan, Poland

**Keywords:** cataract, cataract surgery, follow-up, unexpected management changes

## Abstract

*Background and Objectives:* to evaluate whether a set of questions after a routine cataract surgery can predict unexpected findings and avoid an unnecessary follow-up visit. *Materials and Methods:* single-center, prospective, cohort study included 177 routine cataract surgery cases of two experienced surgeons between November 2019 and December 2020. Inclusion criteria included unremarkable postoperative day one follow-up examination. A set of seven questions regarding complaints with positive or negative answers was presented at the second follow-up visit (PV2)—one week (mean 8.34 ± 1.73 days) after the surgery. The outcome measures were the incidence of unexpected management changes (UMCs) at the PV2 visit (change or addition from a prescribed postoperative drop plan, extra procedures, an urgent referral to an ophthalmologist) and UMCs associations with the answers to a question set. *Results:* 81.4% of patients had no complaints about postoperative ocular status and answered with negative answers, 18.6% reported one or more complaint (positive answer): dissatisfaction with postoperative visual acuity (6.2%, 11 cases), eye pain (4.0%, 7 cases), increase in floaters after the surgery (4.0%, 7 cases), red eye (4.0%, 7 cases) and others. The prevalence of UMCs at PV2 was 1.7% (3 cases), of which 0.6% (1 case) was the prolonged antibiotic prescription due to conjunctivitis, 0.6% (1 case) was the addition of IOP lowering medication and 0.6% (1 case) was additional medication due to uveitis management. None of the complaints (positive answers) at PV2 were associated with the incidence of UMCs (*p* > 0.05). *Conclusions:* there were no associations of UMCs determined with positive answers to the questions. The prediction of UMCs incidence based on the positive answers was not obtained. Thus, we cannot exclude the necessity of a postoperative week one follow-up visit.

## 1. Introduction

Cataracts are the leading cause of blindness affecting about 20 million people and remain an important socioeconomic and public health care problem worldwide with surgery as the only effective treatment, and the standard procedure being phacoemulsification with intraocular lens implantation [1,2,3,4,5,6,7,8,9,10,11].

Cataract surgery is a common day case procedure worldwide, performed 4.5 million times in 2016 on patients with cataracts in European Union member states [12]. With the growing age of the population, the demand for eye care and cataract surgery is expected to double within the next 20 years [1,2,4,5,8,9].

Increasing number of cataract surgeries performed annually means higher postoperative care costs for patients, ophthalmologists, and the entire health care system. Different studies have aimed to determine the most efficient and acceptable postoperative follow–up during early postoperative period. It has been suggested to change and optimize the frequency of postoperative visits due to a low rate of early complications after cataract surgery, with the majority not requiring early surgical intervention. Therefore, the time and number of close postoperative follow-up visits could be adjusted to detect and manage early clinically relevant complications. Such a model can reduce clinical cost, distribute physician time, and improve well-being of the patient assuring recovery after surgery as well as enhance patient satisfaction as well [3,4,5,6,7,8].

A common regimen of the first postoperative visit is based on the 2016 American Academy of Ophthalmology (AAO) Preferred Practice Pattern for Cataract in the Adult Eye which recommends that patients after cataract surgery should be examined on the first postoperative day [13]. The postoperative day one examination is necessary to assess urgent postoperative complications after cataract surgery, while the postoperative month 1 examination is necessary to evaluate postoperative refraction. Nevertheless, postoperative week one review is questionable because of a low rate of unexpected complications [3,4,5,6,7,8,11,13].

The postoperative follow-up situation has become problematic due to the 2019 outbreak of coronavirus disease (COVID-19) and related lockdown [14]. Between March and April 2020, a significant reduction in cataract surgery rates has been reported, reaching up to 97% reduction in some countries [15]. With reopening of the elective ophthalmic service, cataract surgery volume started recovering in the summer of 2020 [16]. As a demand for cataract surgery increased, the importance of managing coronavirus related infection risk remained a priority [17]. Most cataract surgery patients are aged 65 years and older and are considered at a higher risk for worse outcome in cases of Severe Acute Respiratory Syndrome Coronavirus 2 (SARS-CoV-2) infection [18]. Consequently, it is important to re-evaluate patient preoperative and postoperative management to maintain safe conditions for patients and healthcare personnel [14].

There are a few studies about the usage of questionnaires after cataract surgery that could help to detect unexpected complications after cataract surgery [3,10]. In our study, we analyse whether a set of questions after a routine cataract surgery can predict unexpected findings and spare an unnecessary follow-up visit.

## 2. Materials and Methods

The prospective cohort study included routine cataract surgery cases performed by two experienced surgeons between November 2019 and December 2020 and was based on a study by Moustafa et al. [3]. The study was temporarily stopped in March 2020 due to the COVID-19 pandemic related lockdown and resumed in October 2020. All surgeries were performed at a single tertiary center—the Department of Ophthalmology, Lithuanian University of Health Sciences. The study was approved by the Biomedical Research Ethics Committee (Nr. BE-2-26), with an approval date of 9 July 2015. Written informed consent was obtained from all subjects who participated in the study. The study was conducted in accordance with the tenets of the Declaration of Helsinki.

Cataract surgery was considered routine if none of the intraoperative events of complicated cataract surgery occurred: posterior capsule rupture, anterior vitrectomy, placement of a capsular tension ring, extracapsular intraocular lens placement, nuclear fragments in the vitreous body or simultaneous vitreoretinal procedure. Inclusion in the study criteria was an unremarkable postoperative day one follow-up (PV1) examination.

Patients were excluded from the study if any of the following criteria were present at PV1: intraocular pressure higher than 30 mmHg in eyes without glaucoma diagnosis and higher than 21 mmHg in eyes already diagnosed with glaucoma; unexpected clinical findings such as wound leaking, epithelial erosions, corneal oedema, cells in the anterior chamber; addition or a new prescription of intraocular pressure lowering drops or other steroid drops, nonsteroidal or antibiotic drops that differed from the initial surgeon’s postoperative drop prescription; necessity of non-routine postoperative management.

A set of 7 short questions with positive (yes) or negative (no) answers regarding complaints was presented at a second follow-up visit (PV2) first and then ophthalmological examination was performed. PV2 was defined as a visit one week (day range 4–15, mean 8.34 ± 1.73 days) after the surgery. The ophthalmological examination at PV2 was performed by two resident ophthalmologists, but not the surgeons who performed cataract surgeries. The surgeons were not aware of patient answers.

Positive answers included the presence of red eye, increase in floaters after the surgery, eye pain, postoperative vision decrease, presence of new flashes, dissatisfaction with postoperative visual acuity and misunderstanding of postoperatively described eye drop plan. A positive answer was regarded as a probable symptom, which may indicate aetiologies requiring additional treatment.

Routine ophthalmological examination was performed and data from medical records were collected: demographics (gender, age), ocular comorbidities (glaucoma, age-related macular degeneration, previous intravitreal injections, macular hole), and systemic diseases. Non-corrected and best corrected visual acuity (presented in decimal) and intraocular pressure before surgery at PV1 and at PV2 were investigated. Intraocular pressure was measured by air-puff tonometry.

The outcome measures were the incidence of unexpected management changes (UMCs) at PV2 and UMC associations with the answers to a question set. UMCs included change from or in addition to the surgeon’s prescribed postoperative drop plan, extra procedures excluding suture removal, and an urgent referral to an ophthalmologist. The sample size was calculated based on the Paniotto formula.

Statistical analysis was performed with MS Excel 2010 and IBM SPSS 22.0. Basic categorical variable characteristics were presented in percentage (%). Descriptive continuous variable statistical data results were described as mean ± standard deviation (SD). Differences of the categorical variables were assessed with Fisher exact test. Logistic regression analysis was performed to identify the association of the positive answers with the incidence of UMCs. *p* value <0.05 was considered statistically significant.

## 3. Results

Out of 182 routine cataract surgery cases analysed at the postoperative day one follow-up visit (PV1), 177 cases (97.25%) were included in the statistical analysis. Descriptive data is shown in Table 1 and Table 2.

In 81.4% of cases, patients had no complaints about postoperative ocular status and gave only negative answers at PV2. 18.6% reported one or more complaints (positive answer): dissatisfaction with postoperative visual acuity—11 cases (6.2%), eye pain—7 cases (4.0%), increase in floaters after the surgery—7 cases (4.0%), red eye—7 cases (4.0%). Other complaints were reported less frequently: presence of new flashes—4 cases (2.3%), misunderstanding of postoperatively described eye drop plan—2 cases (1.1%) and postoperative vision decrease—1 case (0.6%) (Table 3).

The prevalence of UMCs at PV2 was 3 cases (1.7%), of which 1 case (0.6%) was prolonged antibiotic prescription due to conjunctivitis, 1 case (0.6%) was the addition of IOP lowering medication and referral for further glaucoma evaluation, and 1 case (0.6%) was additional medication due to uveitis management. Other than suture removal, no cases of extra procedures or urgent referral to an ophthalmologist were observed. (Table 4).

None of the complaints (positive answers) at PV2 were associated with the incidence of UMCs (*p* > 0.05).

## 4. Discussion

This study was carried out to evaluate whether a set of questions after routine cataract surgery can predict unexpected findings at postoperative week one examination and avoid unnecessary follow-up visits. We found an extremely low rate of UMC at postoperative week one visit—1.7% (3 cases) of which, 0.6% (1 case) was prolonged antibiotic prescription due to conjunctivitis, 0.6% (1 case) was the addition of IOP lowering medication and referral for further glaucoma evaluation and 0.6% (1 case) was additional medication due to uveitis management. None of the positive answers from the set of questions were associated with the incidence of UMC (*p* > 0.05). Patients with positive answers had none of the signs of an eye disease or surgery complications during the PV2 visit. Positive answers without any significant UMCs were considered as normal postoperative recovery findings related to other existing eye diseases (e.g., dry eye disease or high postoperative visual acuity expectations in amblyopia) that were not related to the surgery.

Moustafa et al. have conducted a similar study (*n* = 254) on optimization of follow-up visits after cataract surgery using a standard question set to predict UMC at the postoperative week one visit. They defined the UMC as a deviation from the eye drop taper plan, such as antibiotic, steroid, intraocular pressure lowering or non-steroidal anti-inflammatory drops prescribed at the first postoperative day visit, an addition of the eye drops excluding artificial tears, performance of a procedure excluding suture removal, or urgent referral to an ophthalmologist. In this analysis, 4.7% (8 cases) of UMCs were found, but the incidence of UMC in cases without positive answers to the question set was zero, and the authors suggested that a postoperative week one follow-up visit was not needed for patients with negative answers to the question set. The study observed that the question set was practical and easy to apply and could also be delivered by phone or social media [3].

A standard questionnaire was also used in another study (*n* = 256), where nurse-administered telephone questionnaire was used at the first postoperative day visit. The nurses presented a questionnaire about patients’ general condition, ocular pain, headache, nausea, vomiting and visual acuity at the postoperative day one visit. Patients with prior symptoms were advised for an immediate ophthalmologist appointment. They determined only one case out of 238 with poor general condition, blurred vision and eye pain that was related to corneal oedema and raised intraocular pressure. This study suggests that the first day postoperative visit can be safely omitted for a nurse-administered telephone survey, which can be an alternative to a postoperative day one visit for detecting early complications after cataract surgery and urgent referral to an ophthalmologist [10].

Some studies have examined complications during early postoperative period to optimize postoperative follow-up visits without the use of a question set. According to some authors, an early postoperative visit is important for detecting intraocular pressure elevation, which is the most frequent postoperative complication and is associated with an early steroid response [3,4,5].

Other researchers have examined unexpected management changes (UMC) rate without a questionnaire in asymptomatic patients one week after uneventful cataract surgery (*n* = 1938). They found only 0.9% (11 cases) of UMC at postoperative week one visit, of which the most common was the addition of IOP lowering drops due to IOP rise. One of those patients had ocular hypertension observed and none of other patients had glaucoma, were glaucoma suspects or had ocular hypertension. There were no cases of endophthalmitis or retinal detachment at the postoperative week one visit. It has been suggested that postoperative week one examination might be omitted for the appropriate subgroup of patients [5].

McKellar et al. have conducted similar research (*n* = 1000) to detect early complications and the necessity of a postoperative week one visit. They included both complicated and uncomplicated histories and cataract surgeries in the study. The complication rate was 4.1% (41 cases), the most common complications were: uveitis, tight sutures, and cystoid macular oedema. Out of these patients, 50% had an unremarkable history until postoperative week one examination. Investigators have noticed that all patients with an identifiable preoperative or intraoperative risk factors should be routinely examined at postoperative week one visit [6].

In the Seed et al. study, patients were examined two hours and two weeks after cataract surgery to investigate the safety of deferring the postoperative visit after phacoemulsification cataract surgery until two weeks after the procedure (*n* = 115). An ophthalmic review two hours after surgery identified 22% (25 cases) of intraocular pressure spike to 30 mmHg or higher and 1.7% (2 cases) of corneal abrasions that required intervention. The investigators referred to asymptomatic IOP rise as being common in the immediate postoperative period, and while the same-day ophthalmic review was important, the first postoperative review could be safely delayed until two weeks after cataract surgery in those cases where asymptomatic or transient IOP spike would not be assumed as clinically important [5]. A similar systematic review and meta-analysis (*n* = 886) has been conducted to identify the safety of deferring the first review after uneventful cataract surgery until two weeks postoperatively. It has been recommended that the first postoperative visit can be deferred until two weeks after surgery for low-risk (non-glaucomatous) patients, while patients with intraoperative complications, glaucoma or being operated on by inexperienced surgeons should be followed closely during the postoperative period [8].

A different methodology was followed by Wesborg and Mönestam (*n* = 1249). They have evaluated the safety perspective when a standard routine after cataract surgery was changed to an unplanned visit after surgery without significant ocular comorbidities and/or surgical complications. Patients with ocular comorbidities, such as glaucoma, wet age-related macular degeneration and/or diabetic maculopathy and patients with intraoperative complications were scheduled for a postoperative visit. Only 9% (117 cases) of 1249 patients initiated a postoperative visit; from those, 26% (30 cases) already had a planned appointment to an ophthalmologist. The most common objective reason was poor visual acuity caused by corneal oedema that could be associated with IOP rise or endothelium insufficiency. It was noticed that elevation of IOP after surgery could be left untreated if it was not associated with corneal oedema or patient discomfort, excluding glaucoma patients who must always have follow-up visits [7].

Despite different methods of postoperative care and follow-up, AAO suggests that the frequency of postoperative visits is based on the goal of optimizing the outcome of surgery and promptly recognizing and managing complications. Nevertheless, postoperative week one review is questionable due to a low rate of unexpected complications [3,4,5,6,7,8,11,13]. The first postoperative visit should be within 24 or 48 h of surgery, considering patient characteristics—intraoperative complications or high risk of immediate postoperative complications (e.g., IOP spike). Further subsequent visits depend on the medical condition of the operated eye, refraction and visual function, and more frequent postoperative visits are indicated if unusual findings, such as symptoms of complications occur. A final visit after uneventful cataract surgery should be made to give an accurate prescription for optical correction to allow for the patient’s optimal visual function [13].

Our study has few limitations. Firstly, the sample size was affected due to COVID-19 pandemic related lockdown, resulting in a significant reduction in cataract surgery rates. A larger cohort, more UMCs and adjustments in methodology may provide different results. Moreover, including more than one tertiary center and more than two surgeons may provide more validity to the study. Lastly, only seven short questions regarding complaints were presented at PV2. A different and more extensive set of questions may be a more suitable and susceptible tool to determine probable UMCs.

Postoperative follow-up visits are a significant workload for eye clinics, which can be reduced by optimizing the frequency of postoperative visits, especially with an unprecedented public health crisis due to COVID-19, and related infection control measures to ensure both patient and staff safety. Standard questions could help to identify patients that need an urgent visit. This model or alternative methods, such as telephone reviews or social media communication, could help rationalize care for both patients and providers, as well as to assure that all possible precautions are being taken to prevent COVID-19 infection.

## 5. Conclusions

In conclusion, there were no associations determined between UMCs and positive answers to the questions in our study, thus we cannot exclude the necessity of a postoperative week one follow-up visit.

## Figures and Tables

**Table 1:** Demographic data and comorbidities ^1^.

Descriptive	*N* (%)/Mean ± SD
Age, years	73.07 ± 8.975
Male age, years	72.65 ± 8.776
Female age, years	73.27 ± 9.098
Male/female gender	57 (32.2)/120 (67.8)
Eyes with/without glaucoma diagnosis	30 (16.9)/147 (83.1)
Eyes with/without ARMD	46 (26.0)/131 (74.0)
Eyes with nonexudative/exudative ARMD	41 (23.2)/5 (2.8)
Eyes previously treated with IVT injections	5 (2.8)
Eyes with macular hole	1 (0.6)
Patients with/without DM	25 (14.1)/152 (85.9)

^1^ Abbreviations: ARMD—age-related macular degeneration, IVT—intravitreal injections, DM—diabetes mellitus, SD—standard deviation.

**Table 2:** Visual acuity and IOP data ^1^.

Descriptive	Mean ± SD	Range
NCVA before surgery, decimal	0.23 ± 0.169	0.00–1.00
BCVA before surgery	0.27 ± 0.202	0.00–1.00
IOP before surgery, mmHg	14.86 ± 2.825	10.00–26.00
NCVA at PV1, decimal	0.62 ± 0.270	0.09–1.00
BCVA at PV1, decimal	0.69 ± 0.245	0.09–1.00
IOP at PV1, mmHg	14.94 ± 4.273	7.00–25.70
NCVA at PV2, decimal	0.74 ± 0.235	0.06–1.00
BCVA at PV2, decimal	0.89 ± 0.203	0.10–1.00
IOP at PV2, mmHg	14.32 ± 3.525	7.00–27.0

^1^ Abbreviations: NCVA—not-corrected visual acuity BCVA—best-corrected visual acuity, IOP—intraocular pressure, PV1-postoperative day one follow-up visit, PV2-second follow-up visit (PV2).

**Table 3:** Incidence of answers to the questions.

Descriptive	*N* (%)
Only negative answers	144 (81.4)
1 or more positive answers	33 (18.6)
1 or more positive answer:	
• Dissatisfaction with postoperative visual acuity	11 (6.2)
• Eye pain	7 (4.0)
• Increase in floaters after the surgery	7 (4.0)
• Red eye	7 (4.0)
• Presence of new flashes	4 (2.3)
• Misunderstanding of postoperatively described eye drop plan	2 (1.1)
• Decreased vision postoperatively	1 (0.6)

**Table 4:** Incidence of unexpected management changes ^1^.

Descriptive	*N* (%)
Suture removal at PV2	1 (0.6)
UMCs:	3 (1.7%)
• Prolonged antibiotic prescription	1 (0.6%)
• Additional IOP lowering medication	1 (0.6%)
• Uveitis management	1 (0.6%)

^1^ Abbreviations: PV2—second follow-up visit, UMCs—unexpected management changes, IOP—intraocular pressure.
